# Highlighting convergent evolution in morphological traits in response to climatic gradient in African tropical tree species: The case of genus *Guibourtia* Benn.

**DOI:** 10.1002/ece3.5740

**Published:** 2019-11-12

**Authors:** Félicien Tosso, Jean‐Louis Doucet, Kasso Daïnou, Adeline Fayolle, Alain Hambuckers, Charles Doumenge, Honoré Agbazahou, Piet Stoffelen, Olivier J. Hardy

**Affiliations:** ^1^ Forest is Life TERRA Teaching and Research Centre Gembloux Agro‐Bio Tech University of Liège Gembloux Belgium; ^2^ Evolutionary Biology and Ecology Unit Faculté des Sciences Université Libre de Bruxelles Brussels Belgium; ^3^ Nature+ asbl, s/c Forest is Life TERRA Teaching and Research Centre Gembloux Agro-Bio Tech University of Liège Gembloux Belgium; ^4^ UR SPHERES Behavioral Biology University of Liege Liege Belgium; ^5^ Centre International de Recherche Agronomique pour le Développement TA C‐105/D, Campus International de Baillarguet Montpellier France; ^6^ Herbarium Botanic Garden Meise Meise Belgium

**Keywords:** evolutionary ecology, *Guibourtia*, niche comparison, phenotypic adaptation, Phylogenetic Independent Contrast, phylogenetic signal, speciation, taxonomy

## Abstract

1. Adaptive evolution is a major driver of organism diversification, but the links between phenotypic traits and environmental niche remain little documented in tropical trees. Moreover, trait‐niche relationships are complex because a correlation between the traits and environmental niches displayed by a sample of species may result from (a) convergent evolution if different environmental conditions have selected different sets of traits, and/or (b) phylogenetic inertia if niche and morphological differences between species are simply function of their phylogenetic divergence, in which case the trait‐niche correlation does not imply any direct causal link. Here, we aim to assess the respective roles of phylogenetic inertia and convergent evolution in shaping the differences of botanical traits and environmental niches among congeneric African tree species that evolved in different biomes.
2. This issue was addressed with the tree genus *Guibourtia* Benn. (Leguminosae and Detarioideae), which contains 13 African species occupying various forest habitat types, from rain forest to dry woodlands, with different climate and soil conditions. To this end, we combined morphological data with ecological niche modelling and used a highly resolved plastid phylogeny of the 13 African *Guibourtia* species.
3. First, we demonstrated phylogenetic signals in both morphological traits (Mantel test between phylogenetic and morphological distances between species: *r* = .24, *p* = .031) and environmental niches (Mantel test between phylogenetic and niche distances between species: *r* = .23, *p* = .025). Second, we found a significant correlation between morphology and niche, at least between some of their respective dimensions (Mantel's *r* = .32, *p* = .013), even after accounting for phylogenetic inertia (Phylogenetic Independent Contrast: *r* = .69, *p* = .018). This correlation occurred between some leaflet and flower traits and solar radiation, relative humidity, precipitations, and temperature range.
4. Our results demonstrate the convergent evolution of some morphological traits in response to climatic factors in congeneric tree species and highlight the action of selective forces, along with neutral ones, in shaping the divergence between congeneric tropical plants.

## Graphical abstract

Here, we aim to assess the respective roles of phylogenetic inertia and convergent evolution in shaping the differences of botanical traits and environmental niches among congeneric African tree species that evolved in different biomes. Our results demonstrate for the first time the convergent evolution of some morphological traits in response to climatic factors in congeneric tree species and highlight the action of selective forces, along with neutral ones, in shaping the divergence between congeneric tropical plants.

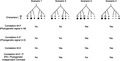

## 1 INTRODUCTION

Historical and environmental factors contribute both to variation in traits across species (Freckleton & Jetz, 2008). In the basic theory of quantitative genetics, a rapid evolutionary change in traits that are selectively sensitive to labile environmental variables is expected (Labra, Pienaar, & Hansen, 2009). But these expectations met complications since it is difficult to discard relative role of environmental determinants and phylogenetic effects in plant species traits variation (Grafen, 1989; Desdevises, Legendre, Azouzi, & Morand, 2003; Diniz‐Filho, de Sant'Ana, & Bini, 1998; Westoby, Leishman, & Lord, 1995; Wiens & Graham, 2005). In modern phylogenetic comparative methods, this problem is particularly complex when related species have related environmental niches (Labra et al., 2009; Losos, 2008; Price, 1997; Wiens & Graham, 2005). It is then crucial to distinguish the similarity between traits of related species and niche that can be attributable to common ancestry (phylogenetic inertia) from similarity to convergent evolutionary change.

**Figure 1:**
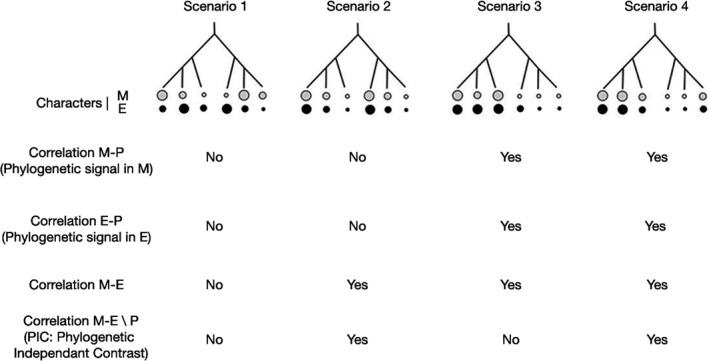
Four hypothetical scenarios (Sc1 to Sc4) regarding the association between a morphological trait (M) and a dimension of the environmental niche (E) among six closely related species of known phylogeny (P). The trait and niche values are represented by the size of each gray (M) or black (E) circle. Under Sc1, species morphological traits and environmental niches evolved quickly, showing no phylogenetic signal, and independently, showing no M‐E correlation. Under Sc2, morphological trait and environmental niche evolved quickly but show strong M‐E correlation due to convergent evolution (same M traits selected in similar E niches). Under Sc3, both morphological trait and environmental niche evolve slowly, leading to phylogenetic signals and resulting in a M‐E correlation, but there is no remaining M‐E correlation when applying a PIC (Phylogenetic Independent Contrast) analysis factoring out the correlation due to phylogenetic dependence because they evolved independently. Under Sc4, a strong M‐E correlation is due both to phylogenetic inertia and convergent evolution (significant PIC analysis). We expect the M‐E correlation to be stronger than the M‐P and E‐P correlations in scenarios 2 and 4 implying convergent evolution, and to be less strong than the M‐P and E‐P correlations in scenario 3 implying only phylogenetic dependence

Species traits can evolve in response to selective pressures but also neutrally (Freckleton & Jetz, 2008). Adaptive processes are suspected when particular traits are associated with particular environmental conditions. However, demonstrating causal links is difficult in macroevolution. To understand what drives the evolution of species traits and niches, phylogenetic comparative methods assess the correlation between characters among species, while accounting for their phylogenetic relationships, to decipher whether trait similarity between species reflects phylogenetic inertia (Figure 1 Sc.3) or convergent evolution (Figure 1 Sc.2; Labra et al., 2009). These approaches have rarely been applied to understand the trait‐niche relationships of tropical trees, partly because well‐resolved phylogenies are often lacking.

Convergent evolution is the independent evolution of a set of similar traits in organisms from different lineages subject to similar abiotic or biotic agents of natural selection (Cody & Mooney, 1978). It is considered as an evidence of adaptation (McLennan & Brooks, 1993; Pagel, 1994) and implies causal traits‐niche relationships. Alternatively to adaptation, phylogenetic inertia has been viewed as taxon‐specific limitations that force a taxon into certain combinations of traits regardless of the niche in which that taxon occurs (Morales, 2000). An apparent trait‐niche association in a set of species may thus occur if the most phylogenetically related species tend to share both similar traits and similar environmental niches due to many possible processes, including genetic drift, stabilizing selection, and constraints.

To date, the mechanisms underlying plant species diversification and the evolution of their morphological traits in tropical forests remain little known. Botanists seek traits that allow distinguishing species or higher taxa but the risk that convergent evolution affects these traits is rarely assessed. Moreover, the role of environmental variation as a potential driver of adaptive morphological evolution (Ortiz‐Medrano, Scantlebury, Vázquez‐Lobo, Mastretta‐Yanes, & Piñero, 2016) has received little attention in African tropical tree species. Understanding these mechanisms can provide important insights into the evolution of African biomes and guide strategies for their conservation (Linder, 2014).

If natural selection by abiotic factors is a main driver of the evolution of species traits, a close relationship between species traits and the environmental conditions can be expected (Ricklefs, 1987; Schluter, 1988). However, this statement remains to be demonstrated, especially for congeneric tropical tree species, because morphologically similar tree species can occur in contrasted environments, for example from dense rain forests to dry woodlands/savannahs, so that the relative importance of adaptive processes in shaping the morphological traits that discriminate them remains unclear.

A comparative approach to explore evolutionary patterns driven by environmental variation (Freckleton & Jetz, 2008) requires a well‐resolved phylogeny of the compared species to distinguish convergent evolution from spurious trait‐niche association due to phylogenetic inertia (Giarla & Esselstyn, 2015). To study the relationships between traits and environmental drivers following an ecomorphological approach (Motta & Kotrschal, 1991; Wiens & Graham, 2005), Broennimann et al. (2012) proposed multivariate techniques to quantitatively compare the environmental niches between pairs of species (e.g., indices of niche overlap, niche equivalency and niche similarity). These new niche modelling tools combined with phylogenetic information (a) help to assess how the realized niches (see Soberón, 2007) of closely related species have evolved over time (e.g., Guisan & Thuiller, 2005; Ortiz‐Medrano et al., 2016), and (b) allow to explain the observed biogeographical patterns and to test ecological hypotheses (Rato et al., 2015).

The present work aimed to understand the relationships between environmental niche and morphological (dis)similarity among congeneric tropical tree species while accounting for phylogenetic inertia. More specifically, we addressed the following questions: (a) Do species morphological traits and environmental niches display phylogenetic signals?, (b) are morphological and environmental (dis)similarity between species correlated?, (c) are morphological and environmental (dis)similarity still correlated after factoring out the impact of phylogenetic inertia suggesting convergent evolution? By answering these questions, we tested whether the association between morphological traits and environmental niche within congeneric species supports one of the four scenarios detailed in Figure 1.

We addressed this issue using the genus *Guibourtia* Benn. (Leguminosae and Detarioideae), which includes 13 African tree species (Tosso, Daïnou, Hardy, Sinsin, & Doucet, 2015). This genus represents a good model for our questions because (a) its species occur over a wide range of habitat types (rainforests, dry forests/savannas), and (b) a well‐resolved phylogeny of this genus is now available (Tosso et al., 2018) and shows three clades, each including species occupying contrasting environments.

## 2 METHODS

### 2.1 Study species

The African *Guibourtia* species occur in a variety of vegetation types, across the Sudano‐Sahelian Region, the Guineo‐congolian Region, and the Zanzibar‐Inhambane regional Mosaic (Tosso et al., 2015). The 13 species can be roughly categorized into species inhabiting relatively dry and seasonal climates harboring tropical dry forests (dense dry forests and woodlands) or wooded savannahs, hereafter identified by the # symbol, and species inhabiting wet/moist and weakly seasonal climates favouring dense wet forests (species with * symbol). Six species are associated with dry and seasonal climates: *G. carrissoana^#^* (M.A.Exell) J.Leonard, *G. coleosperma^#^* (Benth.) Leonard, *G. conjugata^#^* (Bolle) J.Leonard, *G. copallifera^#^* Benn., *G. schliebenii^#^* (Harms) J.Leonard, and *G. sousae^#^* J.Leonard; and seven species to wet/moist climates: G. arnoldiana^*^ (De Wild. & T. Durand) J.Leonard, G. demeusei^*^ (Harms) J.Leonard, G. dinklagei^*^ (Harms) J.Leonard, G. ehie^*^ (A.Chev.) J.Leonard, G. leonensis^*^ J.Leonard, G. pellegriniana^*^ Leonard, and G. tessmannii^*^ (Harms) J.Leonard (Figure S1).

### 2.2 Morphological traits

A total of 281 georeferenced herbarium samples were used for the morphological analyses. They were collected between 1889 and 2010 and conserved in museums and botanical gardens (the National Museum of Natural History in France, the Botanic Garden Meise in Belgium, the Royal Botanic Garden of Edinburg in UK, the Nairobi University Herbarium in Kenya, and the Herbarium of Maputo in Mozambique). Among these samples, 57% were fertile (with flowers, fruits, and/or seeds), and only the specimens for which the determination was validated by experienced botanists in Leguminosae (mostly J. Leonard, J. Wieringa, M. Fougère‐Danezan, or R. Letouzey) were taken into account. A list of 45 morphological characters were measured on all (or only fertile) samples, using the determination keys of Léonard (1950), Aubreville (1970), Tosso et al. (2015) and Fougère‐Danezan, Herendeen, Maumont, and Bruneau (2010) (Table 1). Concerning flowers, once removed, the parts to be measured were rehydrated in boiling water at 90°C for 3 min. The flowers (1–3) were then dissected and observed. The floral pieces were measured with a micrometer incorporated into a binocular microscope (Nachet GLI 154), at magnification ×10–40. The microscope was also used to check for the presence of glands and hairs on the leaflets, flowers, and fruits (Table 1).

**Table 1:** The 45 morphological characters (including 15 vegetative, and 30 reproductive characters) considered for characterizing *Guibourtia* individuals

Morphological traits	Character states or measurement unit in case of quantitative variables
Vegetative characters	
Position of primary leaf veins	Submarginal, marginal, median
Apex leaflets	Obtuse, acuminate
Glands on the abaxial side of the limb	Absent, present
Limb	Membranous, coriaceous, (sub)coriaceous
Petiole hairiness	Glabrous, not glabrous
Gland on petioles	Absent, present
Stipule	Obsolete, persistent
Size of stipules	Absent, tiny, foliaceous
Number of leaflets per leaf	1, 2
Number of secondary leaf veins suprabasilar	1, 2, 3, 4, 5
Number of secondary leaf veins basilar	1, 2, 3
Length of leaflets	cm
Width of leaflets	cm
Length of acumen	cm
Petiole length	cm
Reproductive characters	
Inflorescence position	Axillary, axillary and terminal, terminal
Type of inflorescence	Panicle => cob, cluster
Pedicel	Absent, present
Bracts	Obsolete, persistent
Bracts hairiness	Absent, present
Gland on bracts	Absent, present
Bracteoles	Obsolete, persistent
Shape of bracts	Orbicular, linear
Hairiness of the external face of the bracts	Absent, present
Shape of flower bud	Cylindrical, globular, ellipsoid
Hairiness bud on the external face of the sepals	Absent, present
Hairiness on the inner side of the sepals	Absent, present
Gland on sepals	Absent, present
Hairiness of disc	Absent, present
Pilosity of ovary	Glabrous, pilose
Stipe of the ovary	Sessile, stiped
Hairiness of the stipe's ovary	Glabrous, pilose
Type of fruit	Indehiscent fruit, dehiscent fruit
Gland on fruit	Absent, present
Veins on the fruit outer surface	Absent, present
Stipe of the fruit	Absent, present
Arillus on the seed	Absent, present
Length of sepals	mm
Width of sepals	mm
Length of stipe of the ovary	mm
Length of the fruit	cm
Width of the fruit	cm
Thickness of the fruit	mm
Length of the fruit stipe	cm
Number of seeds per fruit	1, 2

### 2.3 Environmental data

Environmental data corresponding to occurrence points of the 13 *Guibourtia* species were used to model the species environmental niches. To this end, in addition to the geographical coordinates of the 281 specimens used for the morphological characterization, we extracted georeferenced data of reliably determined specimens from Kew herbarium (K) and Naturalis herbarium (L), giving us a total of 401‐presence records after removing duplicates. Climate and soil data were extracted from the Climatic Research Unit (CRU) of the University of East Anglia (Mitchell & Jones, 2005; New, Hulme, & Jones, 1999) and “FAO Digital Soil Map of the World, version 3.6.” Both climatic and soil data were interpolated at 0.5° spatial resolution. To avoid redundancy in environmental information, a principal component analysis (PCA) was used as a data reduction technique (Heikkinen et al., 2006). We chose the least correlated variables that best explain the distribution of *Guibourtia* species: monthly means of temperature (°C), precipitation (mm), solar radiation (w/m^2^), relative humidity (%), temperature range (°C), Potential evapotranspiration (mm), wind speed (m/s), and soil pH.

### 2.4 Phylogenetic data

In a previous study, we built a high‐resolution phylogeny of the genus *Guibourtia* using the whole chloroplast genome (Figure S2, Tosso et al., 2018). We reconstructed the phylogeny and performed molecular dating for the 13 African species using a Bayesian MCMC analysis implemented in BEAST v1.8.2 (Bayesian Evolutionary Analysis by Sampling Trees; Drummond & Rambaut, 2007).

### 2.5 Morphological similarity

To describe the morphological similarity between species, we proceeded in the following steps. First, a morphometric distance matrix between the 281 analyzed specimens was obtained by calculating the Gower distance (Gower, 1971) using the R package “FD” (Laliberté, Legendre, & Shipley, 2014). Second, we applied a PCoA (namely PCoA‐based morphological distance) on this distance matrix to synthesize morphological variation on four axes that explained 85.19% of the total variation. Pearson's correlation coefficients between original morphological variables and axis scores were used to interpret each PCoA axis in terms of morphological gradients. Third, we computed the means and standard errors (SE) of the PCoA scores of the specimens for each species along each axis. Finally, we computed the Euclidean distance between the mean species scores along the first four PCoA axes to describe morphological distances between species.

### 2.6 Environmental niche comparison

The environmental niche of species was described using a principal coordinates analysis, as done for morphological data, to facilitate the analysis of the morphology‐niche relationship. Here, we considered the environmental variables extracted from the 281 specimens for which morphological data were available. We then computed the Gower distance between specimens to perform a PCoA and kept the three first axes explaining 90.10% of the total variation in environmental variables. The mean species scores along these axes were used to describe the environmental niche of the species, and the Euclidean distance between mean species scores along the three axes provided niche distances between species.

We conducted complementary environmental niche comparison analyses using the 401‐presence records to further describe the degree of niche similarity and niche overlap between species, applying the approaches developed by Broennimann et al. (2012). First, to determine the limits of the area occupied by the 401‐presence records (Burgman & Fox, 2003), we applied the α‑hull polygons technique using the R package Alphahull (Pateiro‐López & Rodrıguez‐Casal, 2010). We then obtained the background data (Warren, Glor, & Turelli, 2010) for the 13 species taken together by using a buffer zone of 2,000 km around this area (except in the ocean). The buffer extent was based on preliminary analysis following the method of VanDerWal, Shoo, Graham, and Williams (2009) to optimize the predictive power of the explaining factors. We built several models for each species from a sample, varying buffer extent. Then, the area under the ROC curve (AUC) was plotted as a function of buffer extent and we selected the buffer extent corresponding to the minimal value still producing an appreciable AUC increase. The background was used to perform a smoothing technique described in Broennimann et al. (2012), which implemented an ordination technique (PCA‐ent) that divides the environmental space in cells and applied a kernel density function to determine the “smoothed” density of each species occurrence in each cell.

To determine the degree of shared environmental niche between a pair of species, we calculated an index of niche overlap. This index quantifies the proportion of niche shared by the considered couple of species and can be computed by means of the Schoener's *D* statistic (*D* = 1 – 0.5 ∑ |*p*_X,_ *_i_* − *p*_Y,_ *_i_*| where *p*_X,_ *_i_* and *p*_Y,_ *_i_* stand for the probability assigned by the environmental niche modelling for species X and Y, respectively, to cell *i*; and the sum is taken over all the cells of the bidimensional environmental space; Schoener, 1968; Warren, Glor, & Turelli, 2008). *D* varies from 0 (complete disjunction) to 1 (fully overlapping niches).

The Schoener's *D* statistic was then used to perform two tests of niche differentiation: niche similarity and niche equivalency tests as detailed in Broennimann et al. (2012). The comparison of niche similarity and niche equivalency tests is given in Data S1. All analyses were performed in the R platform (R Development Core Team, 2013).

### 2.7 Testing phylogenetic signal in morphology and environmental niche

We inferred pairwise phylogenetic distance between species [P] (patristic distance; function “cophenetic.phylo” in R package “ape”; Paradis, Claude, & Strimmer, 2004) based on the phylogeny of the *Guibourtia* species. To test the existence of phylogenetic signal (Losos, 2008), we used two methods. First, for univariate data, we computed Blomberg's *K* (Blomberg, Garland, & Ives, 2003) using the function “phylosig” (R package “phytools”; Revell, 2012) for environmental niche and morphology separately using (a) species means of each quantitative morphometric and environmental variable and (b) mean species scores along each morphological and niche PCoA axis. To increase the power of the test, we integrated also the estimated standard errors (SE) of the mean values for each species following Ives, Midford, and Garland (2007). Second, for pairwise distances, we performed Mantel tests (Mantel, 1967) between phylogenetic distance and PCoA‐based morphological or niche distances. We increased the power of the test using the square root of phylogenetic distance matrix (P^1/2^) as suggested by Hardy and Pavoine (2012).

### 2.8 Assessing the morphology—environmental niche relationship

To assess whether environmental niche divergence (PCoA‐based niche distance) and environmental niche overlap (*D* statistic) were related to morphological divergence (PCoA‐based morphological distance), we used Mantel tests. To further assess the morphology‐niche relationship among *Guibourtia* species, we applied two‐block partial least squares (2B‐PLS; Rohlf & Corti, 2000) analysis to explore patterns of covariation between the mean species scores along the same number of PCoA morphological and PCoA environmental niche axes. We retained ten axes to summarize 100% of the total variation in the two datasets. The *Rv* coefficient (Robert & Escoufier, 1976) was used to summarize the amount of covariance in each dataset that is accounted for the other dataset. The significance of all 2B‐PLS summary statistics was assessed via permutation of the rows of each dataset.

### 2.9 Morphological adaptation and niche divergence accounting for phylogenetic inertia

Furthermore, to verify whether the morphology‐niche relationship is not simply due to phylogenetic inertia, we performed two types of analyses. First, partial Mantel test examined whether environmental niche was still related to morphological traits after accounting for phylogenetic relationships (using a matrix of phylogenetic raw distance as covariate). Second, we employed the Phylogenetically Independent Contrasts (PIC) approach (Felsenstein, 1985) to test the correlation between species scores along each of the first two morphological PCoA axes with each of the first two niche PCoA axes. PIC analyses were also done on each morphological trait weighting in the first two niche axes in order to identify the specific morphological traits selected by each niche axis. The null hypothesis for PIC test assumes no evolutionary link between two traits (evolution by purely random genetic drift without selection). All the analyses were performed in the R platform (R Development Core Team, 2013), using the functions “pic” (R package “ape”) and “mantel.partial” (R package “vegan”).

## 3 RESULTS

### 3.1 Morphological variation among species and phylogenetic signal

**Figure 2:**
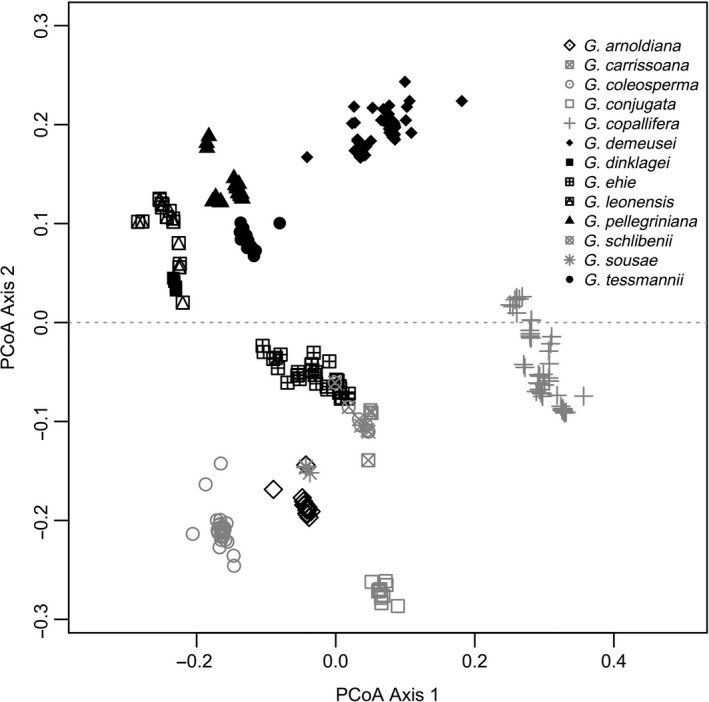
Principal Coordinate Analysis on 45 morphological traits. The symbols in black represent species associated with wet/moist climates whereas those in gray illustrate species associated with dry and seasonal climates

The first two components of the PCoA‐based morphological distance explained 32.50% and 26.40% of the total variation, respectively (Figure 2). The results showed that species associated with wet/moist climates (*G. ehie* and *G. arnoldiana* excepted) were characterized by the combination of the following main characters: large and long leaflets exhibiting marginal venation, subcoriaceous limb, apex of leaflet acuminate, axillary inflorescence, persistent and pilose bracteoles, and no pedicels and no venation on the fruit (Table S1). By contrast, the species associated with dry and seasonal climates showed small leaflets with submarginal venation, coriaceous limb, apex of leaflet obtuse, axillary and terminal inflorescence, obsolete and glabrous bracteoles, and venation on the fruit.

We detected significant phylogenetic signal for six out of 45 morphological traits (Table S1). Overall morphological similarity was significantly correlated to phylogenetic distance (Mantel test: *r* = .24, *p* = .031). This trend was also confirmed when Blomberg's *K* test was performed on PCoA‐based morphological axes: The first axis showed significant phylogenetic signal (*K* = 1.2, *p* = .017) while the second one not (*K* = 0.80, *p* = .130). Among the 11 quantitative morphological traits, only four displayed significant *K* values (number of secondary suprabasilar leaf veins, length of leaflets, petiole length, and width of sepals; Table S1).

### 3.2 Pattern of environmental niche evolution

**Figure 3:**
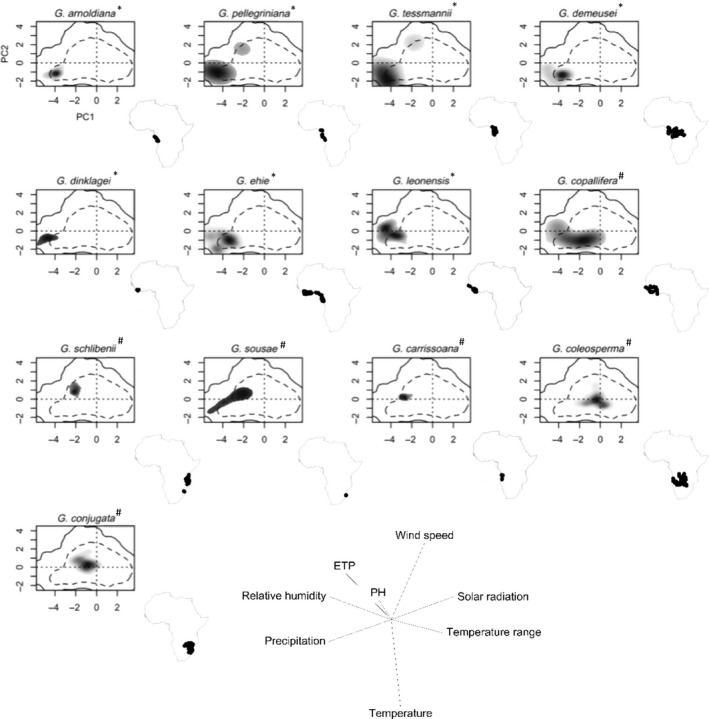
Environmental niches of the 13 African *Guibourtia* species in two main environmental axes produced by the principal component analysis (PCA‐ent) applied on 401 occurrences. For each species, the gray‐to‐black shading represents the grid cell density of the species' occurrences (black being the highest density). The dashed and solid lines delimit respectively 50% and 100% of the available environment conditions in the study area. The last panel presents the contribution of variables for loading the main PCA‐ent axes and the percentage of inertia explained by axes one and two. The geographical distribution of each species is presented below each PCA‐ent

The principal component analysis of environmental niche properties (PCA‐ent) applied on 401 occurrences data gave a first axis explaining 51.7% of the total variance generated by the eight tested variables and mainly loaded by monthly mean thermal amplitude and monthly mean solar radiation toward positive values, and monthly mean potential evapotranspiration and monthly mean precipitation toward negative values (Figure 3). The second axis explained about 19.9% of the variation and was mainly loaded by monthly mean wind speed and pH of the soil toward positive values, and monthly mean temperature toward negative values. All rainforest species showed a distribution in the environmental space with very low values on both PCA‐ent axis 1 (around −4) and axis 2 (around −1, except for *G. leonensis*), which correspond to high precipitation and low solar radiation. Dry forest and woodland species generally displayed highest density at less negative values along axis 1 (between −3 and 0) and tended to differentiate along axis 2 (highest density between −1 and 1 depending on species). The right part of axis 1 (scores > 0) would correspond to the desert zones where *Guibourtia* is absent. Similarly, for axis 2 scores >2 may correspond to low‐temperature areas), inappropriate for the survival and growth of *Guibourtia* species.

As expected, niche overlap values showed that most pairs of rainforest versus dry forest and woodland species occupy different environmental niches (Figure 3, S2). The results highlighted a more marked overlap between the niches of species associated with wet/moist climates than between species associated with dry and seasonal climates as confirmed by the results of niche equivalency (strict niche identity) and niche similarity tests detailed in Data S3.

**Figure 4:**
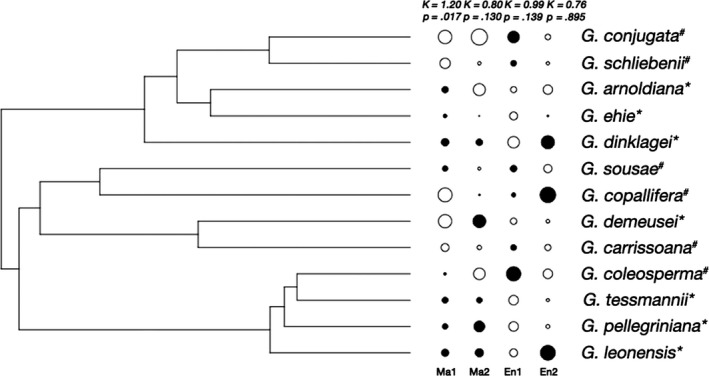
Phylogeny of African *Guibourtia* species with species scores along the first two PCoA axes of morphological data (Ma1 and Ma2) and environmental niche data (En1 and En2)

We detected a phylogenetic signal for species environmental niche through the correlation between phylogenetic distance and PCoA‐based niche distance (Mantel test: *r* = .16, *p* = .04) or niche overlap (Mantel test: *r* = −.23, *p* = .025). However, the univariate Blomberg's *K* tests seemed to lack power (*K* = 0.99, *p* = .139; *K* = 0.76, *p* = .895 for the first two niche PCoA‐based niche distance axes, Figure 4) and when testing each environmental variable in turn, only monthly mean precipitation showed a marginally significant signal (Blomberg's *K* test: *K* = 0.87, *p* = .08; Table S2).

### 3.3 Relationships between morphology and environmental niche without accounting for phylogenetic inertia

We found statistically significant correlations between species morphological similarity and niche overlap (Mantel test: *r* = −.44, *p* < .001) or PCoA‐based niche distance (Mantel test: *r* = .67, *p* = .001). The 2B‐PLS analysis confirmed the strong association between morphology and environment variation: 40% of the covariance in morphology is explained by environmental conditions (*Rv* = 0.40, *p* < .001).

### 3.4 Morphological similarity and environmental niche resemblance accounting for phylogenetic inertia

The partial Mantel test between PCoA‐based niche distance and morphological similarity using phylogenetic distances as a third matrix of covariates was statistically significant (*r* = .24, *p* < .030; Figure 4). This result must be interpreted with caution because partial Mantel tests are liberal (Guillot & Rousset, 2013). Interestingly, when using only quantitative morphological traits, which displayed no significant phylogenetic signal (stipe length of the ovary, thickness of the fruit, length of sepals, length of acumen, and number of secondary leaf veins at the base, Table S1), the relationship between morphological similarity and PCoA‐based niche distance was stronger (Mantel test between species: *r* = .28, *p* = .039) than using only traits that exhibited phylogenetic signal (Mantel test between species: *r* = .12, *p* = .787), supporting that part of the trait‐niche correlation does not result from phylogenetic inertia, as detailed in PIC analyses.

Phylogenetically Independent Contrasts analysis of the four pairwise comparisons between the two first PCoA‐based environmental niche axes and morphological axes was significant only between Axis 2‐morphology and Axis 1‐ environmental niche and marginally significant between Axis 1‐morphology and Axis 2‐ environmental niche (Table 2). The morphological PCoA axis 2 gathered principally flower traits (inflorescence position, type of inflorescence, pedicel, bracts, bracts hairiness, and pilosity on the external face of the sepals of flower buds) and some leaflets and fruit traits (leaflets dimension and venation, veins on the fruit surface) while axis 1 essentially summarized fruit traits (type of fruit, thickness of the fruit, gland on fruit, stipe of the fruit), seeds (arillus on the seed) and some leaflets and flowers traits (number of leaflet veins, aspect of bracteoles, and sepals; Tables S1 and S3). Concerning the environmental niche axes, niche PCoA axis 1 was correlated with solar radiation, relative humidity, precipitations, and temperature range. This axis exhibits rainforest‐dry woodland gradient whereas axis 2 was related to wind speed and potential evapotranspiration.

**Table 2:** Correlation between morphological and environmental niche PCoA axes by phylogenetically independent contrasts

Niche optimum	Axis 1 (solar radiation, relative humidity, precipitations and temperature range)	Axis 2 (wind speed and potential evapotranspiration)
Morphology		
Axis 1 (fruit, seed, leaflet and flower traits)	*r* = −.33; *p* = .290	*r* = −.53; *p* = .075
Axis 2 (flower, leaflet and fruit traits)	***r* = .69; *p* = .018**	*r* = −.44; *p* = .144

The bold values show significance at the 5% level.

## 4 DISCUSSION

In this study, we demonstrated that morphological differences between African *Guibourtia* species are significantly associated with niche divergence, even after factoring out the impact of phylogenetic inertia. This trend was particularly evident when floral traits and leaf dimensions were considered along a rainforest—dry woodland gradient. These findings validate a set of scenarios highlighting (a) both phylogenetic inertia and convergent evolution (scenario 4) and (b) an independent evolution of some morphological traits and environmental niche (scenario 1). They suggest that both selection forces and neutral ones contributed to the morphological divergences and similarities among *Guibourtia* species.

### 4.1 Relationship between morphology, phylogeny, and environmental niche: evidence of adaptive forces

The recent incorporation of phylogenetic and functional information into biogeographical analyses provides a more complete understanding of evolutionary and ecological processes (Molina‐Venegas, Aparicio, Slingsby, Lavergne, & Arroyo, 2015). In this study, six morphological traits, and one morphological PCoA axis representing c. a third of the morphological variation, showed significant phylogenetic signals. This finding is consistent with works of Lee and Collins (2001), Blomberg et al. (2003) and Valverde‐Barrantes, Smemo, and Blackwood (2015) who found that similarity in morphometric forms largely reflects genealogical relationships.

We also proved that some *Guibourtia* morphological traits were correlated with environmental features. This correlation was not only due to phylogenetic inertia, as revealed by three arguments. (a) PIC analysis was significant between two particular M and E axes (PIC: *r* = .69, *p* = .018). (b) The mantel correlation between environmental niche and morphology traits (Mantel test: *r* = −.44, *p* < .001) was stronger than the one between the morphology and phylogeny (Mantel test: *r* = .24, *p* = .031) but also between the phylogeny and environmental niche (Mantel test: *r* = .16, *p* = .04). (c) The morphology‐environmental niche correlation remained significant when the morphological traits exhibiting no phylogenetic signal were considered (Mantel test: *r* = .31, *p* = .008). In fact, the traits significantly correlated to an environmental niche axis did not show phylogenetic signals.

The overall signal between morphology and environmental niche was significant in rainforest species especially when only the reproductive traits were considered. It was also significant for woodland species when all the morphological characters were taken into account together. Among species having dehiscent fruits and arillate seeds dispersed by animals, three are found in rainforests (*G. tessmannii*, *G. pellegriniana*, and *G. leonensis*) and one in woodlands (*G. coleosperma*). Except the latter one, dry forest and woodland species have indehiscent fruits with nonarillate seeds dispersed either by wind or water. This finding was highlighted by Turner (2001) and Chazdon, Careaga, Webb, and Vargas (2003) and confirms the domination of animal dispersed plants in rainforest.

Besides, the relation between PCoA‐based niche distance and morphological dissimilarities was stronger for the PCoA axes considering principally flower traits and leaflets dimension along the rainforest‐dry woodland gradient, which evokes selection forces acting on these phenotypic characters. Rainforest species (e.g., G. tessmannii^*^, G. pellegriniana^*^, G. leonensis^*^ etc.) have longer and larger leaflets than woodland species (*G. sousae^#^*, *G. conjugata^#^*, *G. carrissoana^#^* etc.). As specific leaf area is among the best indicator to assess the adaptation of species to light conditions and water stress for photosynthesis (Hoffmann et al., 2005), a relationship between leave size and environmental niche is expected. Species living in rainforests invest much more in leaf area (adaptation for a competitive light environment) in comparison with species of more open woodlands, while the latter invest more in root development to improve their capture of soil water during the dry season (adaptation to water stress, Hoffmann & Franco, 2003). We also observed that floral traits could be selected. Rainforest *Guibourtia* species have axillary inflorescences while the woodland species have axillary and terminal inflorescences. Herrera (1996) explained this phenomenon by an adaptation to insect pollinators. This kind of adaptation deserves to be more deeply investigated in African biomes.

Even though it is known that each species has specific morphological responses depending on the environmental conditions to which it is subjected to (Gratani, Meneghini, Pesoli, & Crescente, 2003), our results are among the few in Africa that prove that part of the morphological differences between congeneric tree species result from environmental adaptation. This is in line with some studies conducted within various genera, which have found the same association (Cicero & Koo, 2012; Fontanella, Feltrin, Avila, Sites, & Morando, 2012; Fort, Jouany, & Cruz, 2015). Ribeiro, Lloyd, Dean, Brown, and Bowie (2014) also demonstrated that niches differences among species are an evolutionary force shaping diversification, but studies did not clearly demonstrate a direct correlation between morphology and environmental niche. Besides, Couvreur, Porter‐Morgan, Wieringa, and Chatrou (2011) by examining whether speciation was dominated by niche changes found that adaptation to climatic differences between sisters species has not been a major driver of speciation in trees of African tropical rain forests.

However, as shown in our results, the relationship between morphology and environmental niche was not evident for all morphological traits. When some leaflets and flowers traits (number of leaflet veins, aspect of bracteoles and sepals) are considered along the rainforest‐woodland gradient, no significant link was detected between morphology and environmental niche. This result underlines the possible action of genetic drift as an evolutionary force, although one cannot rule out the hypothesis that selection acted again but in response to other factors and climate (Felsenstein, 1985).

### 4.2 Speciation and evolutionary hypotheses within the genus *Guibourtia*

Considering that geographical and ecological factors could contribute to species divergence, two main hypotheses could be proposed to further understand species evolution within the genus *Guibourtia*. The first hypothesis is disruptive ecological adaptation (Knouft, Losos, Glor, & Kolbe, 2006), which assumes that ecological niches tend to diverge in near relatives, reducing interspecific competition (Losos et al., 2003). The second one deals with the fragmentation of favorable habitat of related species described by Acevedo, Melo‐Ferreira, Real, and Alves (2014). This hypothesis supposes that the ancestor of related species occupied a large range that became fragmented in different allopatric refuges following past climatic fluctuations. Tosso et al. (2018) demonstrated that *Guibourtia* started to diversify ~14–24 Myr and continued until Late Miocene and Early Pliocene as confirmed by Fougère‐Danezan (2005). The two first divergence events that occurred ~14.8–16 Myr (Tosso et al., 2018) ultimately lead to three clades corresponding to the subgenera *Gorskia*, *Guibourtia*, and *Pseudocopaiva* described by Léonard (1949). This period coincides with the second major environmental perturbation that occurred from the Early to the Mid‐Miocene (23–15 Mya): Humid vegetation disappeared in the Sahara, the continent moved northward, down positioning the Equator, and the rainforest belt shifted southward (Maley, 1996). During the Late Miocene and Early Pliocene, and especially with the onset of the glacial–interglacial cycles of the Quaternary, the African rainforest probably became fragmented while drier ecosystem types (dry dense forest, woodland, and savannah) expanded (Dupont, Rommerskirchen, Mollenhauer, & Schefuß, 2013). Our results, in combination with the above‐mentioned work, could help hypothesize that the common ancestor of *Guibourtia* species probably occupied a large range in Africa. However, the common ancestor may also have had a restricted area and speciation occurred mainly during geographical expansions.

In addition to the two main hypotheses mentioned previously, it is important to underline three important points. First, each subgenus or clade (Figure S2) presents a wide distribution from West Africa to Southern Africa via Central Africa. Thus, there is no biogeographic signal between these three clades. Second, there is a diversity of configurations between sister species: (a) There are cases of parapatric distribution along environmental gradients that may suggest ecological speciation (*G. demeusei* vs. *G. carrissoana* and *G. coleosperma* vs. *G. tessmannii*), (b) also cases of parapatric to sympatric distribution without strong divergence of niche suggesting allopatric speciation with secondary contact (*G. conjugata*—*G. schliebenii*; *G. arnoldiana*—*G. ehie*), and (c) a case of allopatric distribution, West Africa versus Southern Africa, without strong niche divergence (*G. sousae* vs. *G. copallifera*, but which diverged formerly). Third, it seems that habitat transitions occurred as well from dry environments to moist forest environments (*G. demeusei* based on phylogeny and the dry environment occupied by *G. carrissoana*, *G. copallifera*, and* G. sousae* in the subgenus *Guibourtia*) as in the opposite direction (case of *G. coleosperma* in subgenus *Pseudocopaiva*, and of the clade formed by *G. conjugata* and *G. schliebenii* in the subgenus *Gorskia*). Based on all these observations, it is difficult with the current data to favor a particular mode of speciation within African *Guibourtia* taxa although a certain convergent evolution in morphological traits.

## 5 CONCLUSION

This study is the first in Africa that clearly demonstrated that similar phenotypes evolve independently in different lineages by using new genomic data set and environmental niche modelling techniques with morphological characterization using herbaria collections. We characterized the main environmental variables that constrain the potential distribution of African *Guibourtia* species. We also assessed the diversity and similarity of the environmental niches of these species and demonstrated phylogenetic signals of environmental niche, at some morphological traits and significant correlation between niche divergence and morphological divergence, even after accounting for phylogenetic inertia, at least for some of their respective dimensions. These results are congruent with the scenario 4 (Figure 1, Sc.4) suggest that convergent evolution has occurred. The significant differences in environmental niches spaces also reflect the morphological distances within African *Guibourtia* species due both to neutral processes (e.g., drift) and selection forces at a certain level.

The demonstrated link between morphology and the environmental niche for African *Guibourtia* species could also serve as a basis for predicting long‐term phenotypic changes at the species level in the genus.

## CONFLICT OF INTEREST

None declared.

## AUTHOR CONTRIBUTIONS

F. T., J‐L. D., K. D., and O. J. H. conceived the ideas and designed methodology; F. T., A. H., C. D., and H. A. contributed to the data collection; F. T. analyzed the data under the supervision of J‐L. D. and O. J. H.; and F. T., J‐L. D., K. D., A. F., A. H., C. D., H. A., P. S., and O. J. H. wrote the manuscript. All authors read and approved the final manuscript.

## Data Availability

Data source used to build a high‐resolution phylogeny is cited in the text. Morphological and climate data as well as sampling locations are available via Dryad https://doi.org/10.5061/dryad.cjsxksn1r.
