# Supplementary material for: Highlighting convergent evolution in morphological traits in response to climatic gradient in African tropical tree species: The case of genus Guibourtia Benn
Source: Ecol Evol. 2019 Nov 12;9(23):13114–26. doi: 10.1002/ece3.5740 (PMC6912925; doi:10.1002/ece3.5740)
Supplement: Supplementary file 1 [file ECE3-9-13114-s001.doc]

**Appendices**

**Highlighting convergent evolution in morphological traits in response to climatic gradient in African tropical tree species: the case of genus *Guibourtia* Benn.**

Félicien Tosso^1, 2*^, Jean-Louis Doucet^1^, Kasso Daïnou^3^, Adeline Fayolle^1^, Alain Hambuckers^5^, Charles Doumenge^4^, Honoré Agbazahou^4^, Piet Stoffelen^6^, Olivier J. Hardy^2^

**1.** Central African Forests, TERRA Teaching and Research Centre, Gembloux Agro-Bio Tech, University of Liège, 2 Passage des Déportés, B-5030 Gembloux, Belgium, **2.** Evolutionary Biology and Ecology Unit, Faculté des Sciences, Université Libre de Bruxelles CP160/12, 50 av. F. Roosevelt, 1050 Brussels, Belgium, **3.** Nature+ asbl, s/c Gestion des ressources forestières, Gembloux Agro-Bio Tech, University of Liège, 2 Passage des Déportés, B-5030 Gembloux, Belgium, **4.** Centre international de recherche agronomique pour le développement, TA C-105/D, Campus international de Baillarguet, F-34398 Montpellier cedex 5, France, **5.** UR SPHERES, Behavioral Biology, University of Liege, Belgium, **6.** Herbarium, Botanic Garden Meise, Nieuwe laan 38, 1860, Belgium.

***** **Corresponding Author:** [tfelicien@yahoo.fr](mailto:tfelicien@yahoo.fr)

**Figure S1.** Map of distribution range of African *Guibourtia* species


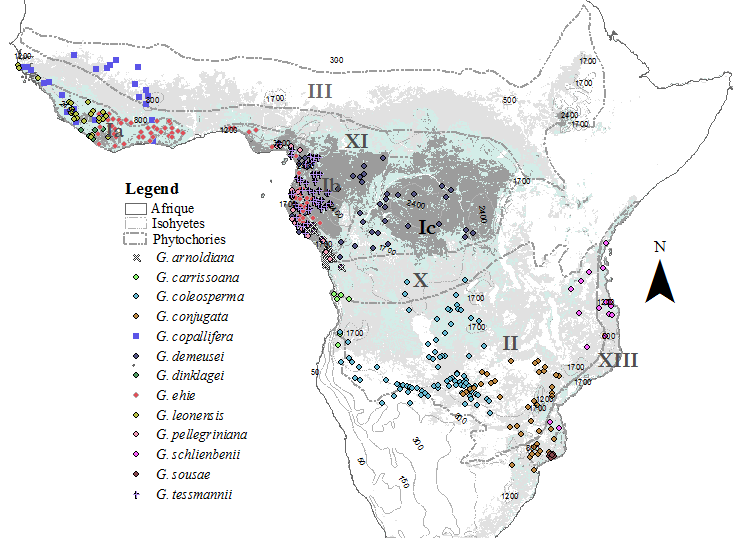


**Figure S2.** High-resolution phylogeny (whole plastic genome sequenced) of African *Guibourtia* species (Tosso et al., submitted) with the subdivisions into subgenera according to Leonard(1949)


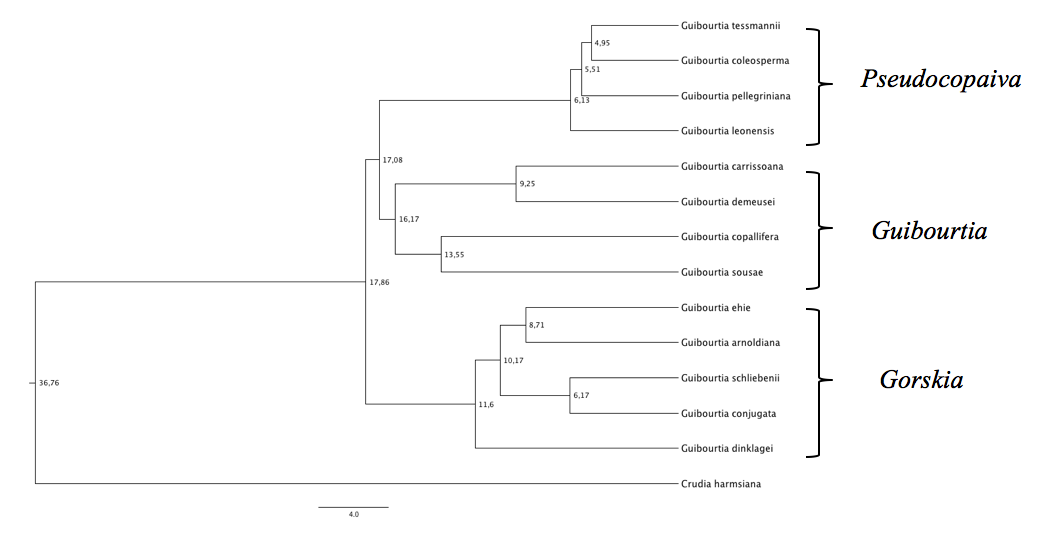


**Table S1.** Correlation of morphological variables with two principal morphology PCoA axes, phylogenetic signal of quantitative morphological traits (Blomber’s K and associated test) and phylogenetic signal test using Maddison and Slatkin (1991) method

| Morphological traits | Axe 1 | Axe 2 | Blomberg’s K | p-value | p-value (phylogenetic signal test; Maddison and Slatkin, 1991) |
| --- | --- | --- | --- | --- | --- |
| Number of leaflets per leaf | -0.11 | -0.05 | - | - | - |
| Number of secondary leaf veins supra basilar | **0.56** | 0.28 | **0.94** | **0.020** | - |
| Number of secondary leaf veins basilar | **-0.88** | 0.17 | 0.53 | 0.239 | - |
| Length of leaflet | 0.1 | **0.68** | **0.73** | **0.053** | - |
| Width of leaflet | 0.07 | **0.52** | 0.64 | 0.174 | - |
| Apex leaflet | -0.27 | **-0.67** | - | - | 0.63 |
| Glands on the underside of the limb | **-0.58** | -0.33 | - | - | 0.165 |
| Length of acumen | 0.06 | 0.33 | 0.62 | 0.338 | - |
| Petiole length | 0.41 | 0.47 | **1.71** | **0.003** | - |
| Petiole hairiness | -0.21 | -0.28 | - | - | 0.397 |
| Stipule | -0.4 | -0.12 | - | - | - |
| Inflorescence position | -0.36 | **-0.82** | - | - | 0.846 |
| Type of inflorescence | 0.18 | **-0.63** | - | - | 0.196 |
| Pedicel | 0.25 | **-0.75** | - | - | 0.42 |
| Bracts | **-0.51** | **0.66** | - | - | 0.365 |
| Bracts hairiness | -0.48 | **0.66** | - | - | - |
| Shape of flower bud | 0.06 | -0.48 | - | - | **0.037** |
| Pilosity of flower bud on the external face of the sepals | -0.34 | **0.58** | - | - | 0.999 |
| Pilosity of calyx on inner side of the sepals | **0.73** | 0.2 | - | - | 0.999 |
| Gland on sepals | **-0.74** | -0.37 | - | - | 0.999 |
| Length of sepals | -0.32 | 0.14 | 0.55 | 0.428 | - |
| Width of sepals | 0.13 | 0.54 | **0.76** | **0.042** | - |
| Pilosity of disc | **0.54** | -0.08 | - | - | 0.999 |
| Pilosity of ovary | **0.72** | 0.01 | - | - | 0.635 |
| Pilosity of the stipe’s ovary | **0.91** | -0.44 | - | - | 0.212 |
| Type of fruit | **-0.75** | -0.03 | - | - | **0.003** |
| Stipe of the ovary | 0.18 | 0.16 | 0.37 | 0.643 | - |
| Gland on fruit | **-0.55** | -0.39 | - | - | 0.401 |
| Thickness of the fruit | **0.55** | 0.2 | 0.62 | 0.21 | - |
| Veins on the fruit surface | -0.02 | **-0.8** | - | - | 0.34 |
| Stipe of the fruit | **0.82** | -0.21 | - | - | 0.389 |
| Length of stipe of the ovary | **0.67** | -0.1 | 0.38 | 0.749 | - |
| Arillus on the seed | **-0.69** | -0.06 | - | - | 0.999 |
| Leaf veins_mediane | -0.11 | -0.05 | - | - | 0.999 |
| Leaf veins_marginale | -0.17 | **0.7** | - | - | 0.999 |
| Leaf veins_marginale | 0.15 | **-0.71** | - | - | 0.999 |
| Limb_coriacous | 0.15 | 0.44 | - | - | 0.133 |
| Limb_membranous | 0.3 | -0.19 | - | - | 0.424 |
| Limb_subcoriacous | -0.11 | **0.54** | - | - | 0.999 |
| Stipule size_absent | -0.4 | -0.12 | - | - | 0.999 |
| Stipule size_tiny | -0.01 | -0.04 | - | - | 0.999 |
| Stipule size_foliaceous | **-0.52** | -0.12 | - | - | 0.999 |

**Table S2.** Correlation of environmental variables with two principal ecological niche optimum axes

|  | **Axis 1** | **Axis 2** | **Blomberg’s K** | **p** |
| --- | --- | --- | --- | --- |
| **Wind speed** | -0.222 | **-0.917** | 0.70 | 0.386 |
| **Temperature** | 0.251 | -0.199 | 0.71 | 0.300 |
| **Solar radiation** | **-0.957** | -0.152 | 0.77 | 0.197 |
| **Relative Humidity** | **0.929** | 0.052 | 0.77 | 0.161 |
| **Precipitation** | **0.787** | -0.335 | **0.87** | **0.085** |
| **Potential evapotranspiration** | 0.014 | **-0.958** | 0.63 | 0.494 |
| **Temperature range** | **-0.935** | 0.075 | 0.61 | 0.630 |
| **pH** | -0.323 | 0.290 | 0.63 | 0.348 |

**Table S3.** Phylogenetically independent contrasts tests on quantitative morphological variables with the two first ecological niche axes

| **Morphological traits** | **Phylogenetically independent contrasts** | | | |
| --- | --- | --- | --- | --- |
|  | Niche Axis 1 | | Niche Axis 2 | |
|  | r | p-value | r | p-value |
| Number of secondary leaf veins supra basilar | -0.346 | 0.270 | 0.449 | 0.143 |
| Number of secondary leaf veins basilar | 0.162 | 0.616 | 0.020 | 0.952 |
| **Length of leaflet** | 0.495 | 0.540 | 0.257 | 0.420 |
| **Width of leaflet** | 0.747 | **0.005** | -0.653 | **0.021** |
| **Length of acumen** | 0.800 | **0.002** | -0.640 | **0.025** |
| Length of sepal | 0.485 | 0.110 | -0.634 | **0.027** |
| Width of sepal | 0.440 | 0.153 | -0.372 | 0.234 |
| **Fruit form** | 0.630 | **0.028** | -0.554 | **0.062** |
| Thickness of the fruit | 0.253 | 0.428 | -0.275 | 0.387 |
