# Supplementary material for: Highlighting convergent evolution in morphological traits in response to climatic gradient in African tropical tree species: The case of genus Guibourtia Benn
Source: Ecol Evol. 2019 Nov 12;9(23):13114–26. doi: 10.1002/ece3.5740 (PMC6912925; doi:10.1002/ece3.5740)
Supplement: Supplementary file 2 [file ECE3-9-13114-s002.doc]

**Supporting information**

**Highlighting convergent evolution in morphological traits in response to climatic gradient in African tropical tree species: the case of genus *Guibourtia* Benn.**

Félicien Tosso^1, 2*^, Jean-Louis Doucet^1^, Kasso Daïnou^3^, Adeline Fayolle^1^, Alain Hambuckers^5^, Charles Doumenge^4^, Honoré Agbazahou^4^, Piet Stoffelen^6^, Olivier J. Hardy^2^

**1.** Central African Forests, TERRA Teaching and Research Centre, Gembloux Agro-Bio Tech, University of Liège, 2 Passage des Déportés, B-5030 Gembloux, Belgium, **2.** Evolutionary Biology and Ecology Unit, Faculté des Sciences, Université Libre de Bruxelles CP160/12, 50 av. F. Roosevelt, 1050 Brussels, Belgium, **3.** Nature+ asbl, s/c Gestion des ressources forestières, Gembloux Agro-Bio Tech, University of Liège, 2 Passage des Déportés, B-5030 Gembloux, Belgium, **4.** Centre international de recherche agronomique pour le développement, TA C-105/D, Campus international de Baillarguet, F-34398 Montpellier cedex 5, France, **5.** UR SPHERES, Behavioral Biology, University of Liege, Belgium, **6.** Herbarium, Botanic Garden Meise, Nieuwe laan 38, 1860, Belgium.

***** **Corresponding Author:** [tfelicien@yahoo.fr](mailto:tfelicien@yahoo.fr)

**Data S1. Comparison niche equivalency and niche similarity tests**

The niche equivalency test was performed by comparing the observed niche overlap value (*D*), using the occurrence of the target species pair, to a null distribution of 100 overlap values obtained after random permutation of the occurrence records between species. We declared non-equivalence of ecological niches if the observed niche overlap value was significantly lower than the null distribution (P ≤ 0.05; Broennimann et al., 2012). Niche equivalency test is a strict test of niche identity (Kirchheimer et al., 2016), assessing whether the environmental ranges of species are interchangeable. Besides, the niche similarity test differs from niche equivalency test since it assesses whether the observed niche overlap value between target species differs from simulated niche overlaps where the geographical distribution of one of the two species is shifted in the background area while keeping the same shape and size (Warren et al., 2010; Broennimann et al., 2012). This asymmetric test compares the niche overlap of one range randomly distributed over its background keeping the range of the other species unchanged (a->b), and then does the reciprocal comparison (a<-b) (Silva et al., 2014). We repeated each randomization 100 times, producing a null distribution of overlap values to which the observed score was compared. This test aims to discern niche differentiation caused by spatial autocorrelation of environmental data and limited species distributions from true ecological niche differences (Broennimann et al., 2012; Hu et al., 2016).

**Data S2. Environmental niche comparisons for African *Guibourtia* species. Niche overlap values are presented for the comparisons of niche similarity and equivalency of species a with species b.**

| *Guibourtia* species | | Niche Overlap | Niche similarity | | Niche Equivalency |
| --- | --- | --- | --- | --- | --- |
| *a* | *b* |  | *a -----> b* | *b -----> a* |  |
| *G. arnoldiana* | *G. pellegriniana* | 0.17 | Similar* | ns | Different* |
|  | *G. tessmannii* | 0.18 | ns | ns | Different* |
|  | *G. demeusei* | 0.22 | ns | ns | Different* |
|  | *G. dinklagei* | 0.14 | ns | ns | Different* |
|  | *G. ehie* | 0.53 | Similar* | ns | Different* |
|  | *G. leonensis* | 0.16 | ns | ns | Different* |
|  | *G. copallifera* | 0.26 | Similar* | ns | Different* |
|  | *G. schliebenii* | 0.3 | ns | ns | Different* |
|  | *G. sousae* | 0.13 | ns | ns | Different* |
|  | *G. carrissoana* | 0 | ns | ns | Different* |
|  | *G. coleosperma* | 0.1 | ns | ns | Different* |
|  | *G. conjugata* | 0.33 | Similar* | ns | Different* |
| *G. pellegriniana* | *G. tessmannii* | 0.83 | Similar* | Similar* | **Equivalent** |
|  | *G. demeusei* | 0.47 | Similar* | ns | Different* |
|  | *G. dinklagei* | 0.56 | Similar* | Similar* | **Equivalent** |
|  | *G. ehie* | 0.32 | ns | ns | Different* |
|  | *G. leonensis* | 0.72 | ns | ns | Different* |
|  | *G. copallifera* | 0.18 | ns | ns | Different* |
|  | *G. schliebenii* | 0.01 | ns | ns | Different* |
|  | *G. sousae* | 0.01 | ns | ns | Different* |
|  | *G. carrissoana* | 0 | ns | ns | Different* |
|  | *G. coleosperma* | 0 | ns | ns | Different* |
|  | *G. conjugata* | 0 | ns | ns | Different* |
| *G. tessmannii* | *G. demeusei* | 0.52 | ns | Similar* | Different* |
|  | *G. dinklagei* | 0.24 | ns | ns | Different* |
|  | *G. ehie* | 0.37 | ns | ns | Different* |
|  | *G. leonensis* | 0.65 | Similar* | Similar* | Different* |
|  | *G. copallifera* | 0.13 | ns | ns | Different* |
|  | *G. schliebenii* | 0 | ns | ns | Different* |
|  | *G. sousae* | 0.01 | ns | ns | Different* |
|  | *G. carrissoana* | 0 | ns | ns | Different* |
|  | *G. coleosperma* | 0 | ns | ns | Different* |
|  | *G. conjugata* | 0 | ns | ns | Different* |
| *G. demeusei* | *G. dinklagei* | 0.45 | ns | Similar* | Different* |
|  | *G. ehie* | 0.6 | ns | ns | Different* |
|  | *G. leonensis* | 0.22 | ns | ns | Different* |
|  | *G. copallifera* | 0.52 | Similar* | Similar* | Different* |
|  | *G. schliebenii* | 0 | ns | ns | Different* |
|  | *G. sousae* | 0 | ns | ns | Different* |
|  | *G. carrissoana* | 0 | ns | ns | Different* |
|  | *G. coleosperma* | 0 | ns | ns | Different* |
|  | *G. conjugata* | 0 | ns | ns | Different* |
| *G. dinklagei* | *G. ehie* | 0.63 | Similar* | Similar* | Different* |
|  | *G. leonensis* | 0.68 | Similar* | Similar* | **Equivalent** |
|  | *G. copallifera* | 0.46 | ns | Similar* | Different* |
|  | *G. schliebenii* | 0 | ns | ns | Different* |
|  | *G. sousae* | 0 | ns | ns | Different* |
|  | *G. carrissoana* | 0 | ns | ns | Different* |
|  | *G. coleosperma* | 0 | ns | ns | Different* |
|  | *G. conjugata* | 0 | ns | ns | Different* |
| *G. ehie* | *G. leonensis* | 0.36 | ns | Similar* | Different* |
|  | *G. copallifera* | 0.32 | ns | ns | Different* |
|  | *G. schliebenii* | 0.1 | ns | ns | Different* |
|  | *G. sousae* | 0 | ns | ns | Different* |
|  | *G. carrissoana* | 0.33 | Similar* | ns | Different* |
|  | *G. coleosperma* | 0 | ns | ns | Different* |
|  | *G. conjugata* | 0.2 | ns | ns | Different* |
| *G. leonensis* | *G. copallifera* | 0.27 | Similar* | ns | Different* |
|  | *G. schliebenii* | 0 | ns | ns | Different* |
|  | *G. sousae* | 0 | ns | ns | Different* |
|  | *G. carrissoana* | 0 | ns | ns | Different* |
|  | *G. coleosperma* | 0 | ns | ns | Different* |
|  | *G. conjugata* | 0 | ns | ns | Different* |
| *G. copallifera* | *G. schliebenii* | 0.1 | ns | ns | Different* |
|  | *G. sousae* | 0.11 | ns | ns | Different* |
|  | *G. carrissoana* | 0.11 | ns | ns | Different* |
|  | *G. coleosperma* | 0.16 | ns | ns | Different* |
|  | *G. conjugata* | 0.13 | ns | ns | Different* |
| *G. schliebenii* | *G. sousae* | 0.58 | Similar* | Similar* | Different* |
|  | *G. carrissoana* | 0.15 | ns | ns | Different* |
|  | *G. coleosperma* | 0.09 | ns | ns | Different* |
|  | *G. conjugata* | 0.67 | Similar* | ns | Different* |
| *G. sousae* | *G. carrissoana* | 0.33 | Similar* | ns | Different* |
|  | *G. coleosperma* | 0.11 | ns | ns | Different* |
|  | *G. conjugata* | 0.53 | ns | ns | Different* |
| *G. carrissoana* | *G. coleosperma* | 0.13 | ns | ns | Different* |
|  | *G. conjugata* | 0.39 | Similar* | ns | Different* |
| *G. coleosperma* | *G. conjugata* | 0.43 | Similar* | Similar* | Different* |

ns, not similar.

*The ecological niches are significantly (P < 0.05) more similar than expected by random.

**Data S3. Environmental niche comparison**

For all pairwise comparisons, the null hypothesis of niche equivalency (strict niche identity) was rejected except for the following pairs of rainforest species: *G. pellegriniana^*^* - *G. dinklagei^*^*, *G. tessmannii^*^* - *G. dinklagei^*^*, *G. tessmannii^*^* - *G. ehie^*^* and *G. dinklagei^*^* – *G. leonensis^*^* (Table 2). On the other hand, analyses of niche similarity (Table 2) pointed out significant similarity both for rainforest species pairs (e.g. *G. pellegriniana^*^* - *G. tessmannii^*^*, *G. pellegriniana^*^* - *G. leonensis^*^*, *G. tessmannii^*^* - *G. ehie^*^*, and *G. dinklagei^*^* - *G. leonensis^*^*) and for dry forest and woodland pairs of species (e.g. *G. sousae^#^* - *G. conjugata^#^, G. schliebenii^#^ - G. sousae^#^*). Some pairs (both rainforest and dry forest and woodland pairs of species) shared niche spaces that were more similar than expected by chance but only in one direction (e.g., *G. arnoldiana^*^* - *G. pellegriniana^*^*, and *G. schliebenii^#^* - *G. conjugata^#^*), exhibiting for instance that the niche space of *G. arnoldiana* is included in the one of *G. pellegriniana* but not the reverse because the latter species has a large niche amplitude.

**Data S4. Niche overlap, equivalency and similarity**

A comparison of niches of pairs of African *Guibourtia* species showed low and high values of niche overlap that can be used to interpret the morphological differentiation patterns. The low values of pairwise niche overlap could be explained when the spatial potential distributions (Appendix 5) for pairs of rainforest vs woodland species are compared. As explained by Acevedo et al. (2014), if the rainforest species cannot adapt to suboptimal environmental conditions of dry woodland and savannah, then the barrier will continue to prevent gene flow between these species, and they will inevitably continue to diverge according to the allopatric speciation model, although regular gene flow may occur in the contact zones (Kozak and Wiens, 2006). We observed that 52.38% of pairs of rainforest species occupied niches which exhibited some kind of similarity according to similarity test (23.81% according to niche equivalency test), while only 26.67% of pairs of dry forest and woodland species occupied similar niches according to similarity test (0% according to equivalency test). This result suggests that dry forests and woodlands could have a larger diversity of habitat types and environmental constraints than in rain forests (Favier et al., 2012).

Our results also indicated very few cases of species pairs exhibiting equivalent niche (three pairs of rain forest species). Niche equivalency and niche similarity tests are not always congruent because niche equivalency implies niche similarity but not the reverse. Niche similarity means that two species are more similar than a random expectation, not that they are strictly equivalent. This finding is congruent with what is expected for environmental niches of closely related species that won’t be equivalent but more similar (Warren et al., 2014). Moreover, niche equivalency test is more conservative than the niche similarity test (Aguirre-Gutiérrez et al., 2015) since the former is more sensitive to the geographic location of species pairs.
